# Neonatal Gut Mycobiome: Immunity, Diversity of Fungal Strains, and Individual and Non-Individual Factors

**DOI:** 10.3390/life14070902

**Published:** 2024-07-19

**Authors:** Alexandra Mpakosi, Rozeta Sokou, Martha Theodoraki, Christiana Kaliouli-Antonopoulou

**Affiliations:** 1 Department of Microbiology, General Hospital of Nikaia “Agios Panteleimon”, 18454 Piraeus, Greece; 2 Neonatal Intensive Care Unit, General Hospital of Nikaia “Agios Panteleimon”, 18454 Piraeus, Greece; anastasiosmmr@yahoo.gr; 3 Neonatal Department, National and Kapodistrian University of Athens, Aretaieio Hospital, 11528 Athens, Greece; 4 Department of Immunology, General Hospital of Nikaia “Agios Panteleimon”, 18454 Piraeus, Greece; c.kalanto@gmail.com

**Keywords:** microbiome, mycobiome, microbes, neonates, immune system, neonatal infection, neonatal fungemia

## Abstract

The human gastrointestinal ecosystem, or microbiome (comprising the total bacterial genome in an environment), plays a crucial role in influencing host physiology, immune function, metabolism, and the gut–brain axis. While bacteria, fungi, viruses, and archaea are all present in the gastrointestinal ecosystem, research on the human microbiome has predominantly focused on the bacterial component. The colonization of the human intestine by microbes during the first two years of life significantly impacts subsequent composition and diversity, influencing immune system development and long-term health. Early-life exposure to pathogens is crucial for establishing immunological memory and acquired immunity. Factors such as maternal health habits, delivery mode, and breastfeeding duration contribute to gut dysbiosis. Despite fungi’s critical role in health, particularly for vulnerable newborns, research on the gut mycobiome in infants and children remains limited. Understanding early-life factors shaping the gut mycobiome and its interactions with other microbial communities is a significant research challenge. This review explores potential factors influencing the gut mycobiome, microbial kingdom interactions, and their connections to health outcomes from childhood to adulthood. We identify gaps in current knowledge and propose future research directions in this complex field.

## 1. Introduction

Several studies have highlighted the importance of the gut microbiome in human health. The human gastrointestinal system contains trillions of microorganisms that coexist in balance, interacting with each other as well as with their host, to regulate metabolism and protect the body from the threat of pathogenic microbes. In normal situations, *Bacteroides* and *Firmicutes* represent the main phyla in the gut microbiome [1]. Nevertheless, species diversity has a key role in maintaining balance between them, avoiding the prevalence of pathogens and the subsequent manifestation of disease. More specifically, species with different functions and ways of reacting to various alterations contribute to microbiome stability, as, in this way, no microbe is allowed to excessively grow to the detriment of others in the intestinal community. On the contrary, the cancelation of species diversity promotes the imbalance between them, gut microbiome destabilization (dysbiosis), the prevalence of pathogens, and finally the manifestation of disease [2].

Moreover, the outer mucus layer of the intestinal mucosal barrier harbors the entire gut microbiome, effectively preventing the harmful microorganisms’ extravasation. However, a rupture in the intestinal barrier dramatically affects the gut–vascular interface, allowing the passage and subsequent dissemination of pathogens and their respective products to peripheral sites of the body, mainly through the portal vein circulation [3]. 

The gut microbiome affects the immune system through the pathogen-associated molecular patterns (PAMPs) antigen complex. Furthermore, during digestion of a rich diet of dietary fibers, the gut microbiome produces short-chain fatty acids (SCFAs), which contribute to the maintenance of intestinal integrity as well as the inhibition of inflammation and carcinogenesis, mainly by activating immunity and gene expression [4]. The SCFAs also improve metabolism by decreasing lipolysis while increasing fatty acid oxidation and glucose-stimulated insulin secretion. In contrast, in a dysbiotic intestinal environment, pathogenic microorganisms, mainly Gram-negative bacteria, prevail, acting by their lipopolysaccharide (LPS) on Toll-like receptors (TLRs), subsequently promoting the inflammatory process by activating several pro-inflammatory cytokines (interleukins: IL-1β, IL-18, IL-6, IL-33, tumor necrosis factor alpha (TNFα), and interferon gamma (IFNγ)), increasing intestinal permeability and pathogen dissemination via the circulation [5,6]. The consumption of processed foods, use of drugs, sedentary life, stress, hyper-disinfection conditions, etc. damage the species diversity of the gut microbiome, triggering the above cascade of inflammation. 

The colonization of the human intestine by microbes in the first two years of life determines the subsequent composition and species diversity, affecting the immune system and health in adulthood. Higher levels of exposure to pathogens in infancy are a key determinant of immunological memory and acquired immunity [7]. On the contrary, the upbringing of children in low socioeconomic conditions, as well as in large families, and the frequent exposure to pathogens cause the development of immunological memory and strong defense mechanisms against infections, allergies, and autoimmune diseases. Moreover, several factors before, during, and after birth have been implicated in the development of gut dysbiosis (maternal health habits and status, mode of delivery, minimal breastfeeding duration, etc.) [8]. 

Most studies that have analyzed the factors influencing the composition and diversity of the gut microbiome have focused mainly on the bacterial component of the gut, neglecting the fungi that, although less abundant, play an important role in human health and disease [9]. They also play a key role for newborns, who, due to their immature immune system and intestinal barrier, are vulnerable to life-threatening infections and the development of chronic diseases later in life. However, the development of the gut mycobiome in infants and children remains poorly understood. There are limited data on the early-life factors influencing the composition of the gut mycobiome. Furthermore, understanding how these factors interplay with microbial communities during critical developmental windows remains a significant research challenge. In this review, we explore the potential factors influencing the gut mycobiome, interactions between different microbial kingdoms, and connections to health outcomes throughout childhood and adulthood. Additionally, we identify gaps in current knowledge of this complex ecosystem and propose future directions for research in this area.

## 2. Immune Response against Fungi

It has recently been shown that certain fungal species can interact with the immune system through molecular patterns that activate innate immune receptors. Some fungi, including *Aspergillus fumigatus*, *Candida albicans*, and *Malassezia* spp., can activate antigen-specific T-lymphocyte responses just as bacteria or viruses do [10]. Antigen-specific T helper cells contribute to the body’s defense against fungi, even during fungal colonization of the skin and gut. In particular, the activation of Th17 protects against commensal fungi such as *C. albicans*. This activation contributes to the maintenance of the integrity of the epithelial barrier and the production of antimicrobial peptide (AMP), which helps to maintain the homeostasis of the microbiome through interaction and balance between bacterial and fungal communities. It has been hypothesized that this Th17 specialization for *C. albicans* may be due both to the particular characteristics of this yeast and to its long-term presence in the gastrointestinal tract, even from the very first days of human life. It is also characteristic that these *C. albicans*-specific Th17 cells can have cross-reactions against other species and heterologous antigens [11,12]. Moreover, this activation of Th17 cells is mostly induced during *C. albicans* colonization without the presence of inflammation [13,14]. In addition, it seems that Th17 cells further prepare the host defense against potential impending tissue damage and fungal invasion by promoting neutrophil recruitment and activation [15,16]. 

Furthermore, the cells of the immune system, such as dendritic cells, macrophages, and natural killer T cells, recognize through their receptors (pattern recognition receptors, PRRs) specific fungal molecular patterns (pathogen/microbe-associated molecular patterns, PAMPs) such as cell wall polysaccharides (mannan, β-glucan, chitin, α-glucan, galactomannan, and galactosaminogalactan) and promote opsonization by complement and antibodies [17,18]. 

The immune system of newborns depends almost exclusively on the immune status of the mother. Newborns are mainly protected from pathogens by maternal immune cells and antibodies transferred through the placenta, such as IgG (Immunoglobulin G) (mainly during the third trimester of pregnancy), or through breastfeeding, such as IgA and IgG [19,20]. Breast milk, in particular, contains immune cells such as monocytes/macrophages, granulocytes and lymphocytes, NK cells, and epithelial cells [21]. 

Transfer of IgG through the placenta contributes to complement activation and phagocytosis of microorganisms in the neonate. This depends on the mother’s total IgG level, on the gestational age (IgG transfer through the placenta begins at the 15th week of gestation and peaks in the third trimester, with adverse consequences for premature infants), the state of the placenta, and the IgG subclass (IgG1, IgG2, IgG3, and IgG4) [19,22]. For example, maternal IgG1 has been shown to provide protection to the fetus and newborn from antitoxins and viral proteins, and IgG1, IgG2, and IgG3 from bacteria [21,23,24]. However, when the mother has an autoimmune disease, autoantibodies can also be transferred through the placenta and harm the newborn, causing neonatal lupus erythematosus, neonatal pemphigus, neonatal epidermolysis, and idiopathic thrombocytopenic purpura [25,26]. Maternal IgA provides immunity to the newborn through mainly intracellular neutralization of adhesins and toxins and blocking of bacterial adhesion [27,28]. Maternal microchimeric immune cells such as T-lymphocytes, PAMMs (placenta-associated maternal macrophages), NK cells, ILCs (innate lymphoid cells), and DCs (dendritic cells) are also transferred through the placenta and contribute to immune tolerance and the replenishment of immunodeficiency [21,29,30]. 

During pregnancy, the neonatal gut microbiome can be influenced by the maternal microbiome, which is gradually colonized first by facultative anaerobes (*Enterobacteria* and *Lactobacilli*) and then by anaerobes (such as *Bifidobacterium*, *Bacteroides*, *Clostridium*, and *Eubacterium*) [31]. The formation of the neonatal microbiome is influenced by factors related to pregnancy and delivery (gestational age, mode of delivery, and maternal condition), genetic background, type of feeding, and the use of antibiotics. The use of antibiotics during the neonatal period can profoundly impact the development of newborns’ microbiome. While antibiotics are essential for treating infections in neonates, they have been implicated in disturbing the delicate balance of gut bacteria, resulting in alterations in microbial diversity and composition. This disruption, known as dysbiosis, has the potential to hinder the maturation of the immune system and increase the risk of certain health issues later in life [32,33]. Any condition that increases prenatal stress has been shown to lead to maternal dysbiosis, which is associated with increased IL-1β in utero, a decrease in BDNF (brain-derived neurotrophic factor), and further long-term effects on behavior and the offspring microbiome [32,34,35]. The newborn is colonized during vaginal delivery with microorganisms from the mother’s vagina and intestine and via cesarean section with microorganisms from the maternal skin and the hospital environment. This is how the neonatal gut microbiome is initially formed, with consequences for the infant’s metabolism and health status for months and perhaps even years [36,37]. The birth via cesarean section causes a delay in the formation and diversity of the gut microbiome during the first two years of life, as well as a delay in the development of immune cells such as regulatory T cells and in the maturation of the intestinal epithelium and mucus layer [38,39]. Neonatal gut microbial colonization affects the development and maturation of the immune system, as commensal bacteria seem to be involved in the development of lymphoid structures in the gastrointestinal tract [21,40]. 

As mentioned above, in healthy individuals with an intact immune system, intestinal colonization with *C. albicans* rarely leads to invasive infection. This appears to be due to the Th17 CD4+ T cell response and subsequent stimulation by IL-17 through the accumulation and activation of circulating neutrophils that further enhance IL-17 secretion. However, immunosuppression and, in particular, neutropenia predispose to the development of candidemia [41]. It has also been argued that the origin of candidemia is related to *candida* species. Isolation in blood cultures of *Candida parapsilosis* may indicate a skin origin of candidemia, while *Candida albicans* may indicate a gut origin. *Candida* species can be introduced into the bloodstream either by lysis of the skin with central venous catheters or from the gastrointestinal tract through the intestinal barrier [42,43]. 

Unfortunately, premature neonates are unable to defend themselves against fungal overgrowth. Low mucin production in preterm infants allows extensive colonization of the mucosal epithelium by fungal hyphae. In addition, premature infants often exhibit decreased production of antimicrobial peptides and impaired neutrophil numbers and function [44]. The response of immature monocytes and macrophages usually occurs in an exaggerated, hyperinflammatory manner [45]. Pattern recognition receptors (PRRs) are either functionally immature due to prematurity or overactivated, causing inflammation. Intestinal sIgA (secretory immunoglobulin A) in preterm infants is also insufficient, while very low birth weight (VLBW) preterm infants cannot even replenish it from breast milk [46].

**Figure 1:**
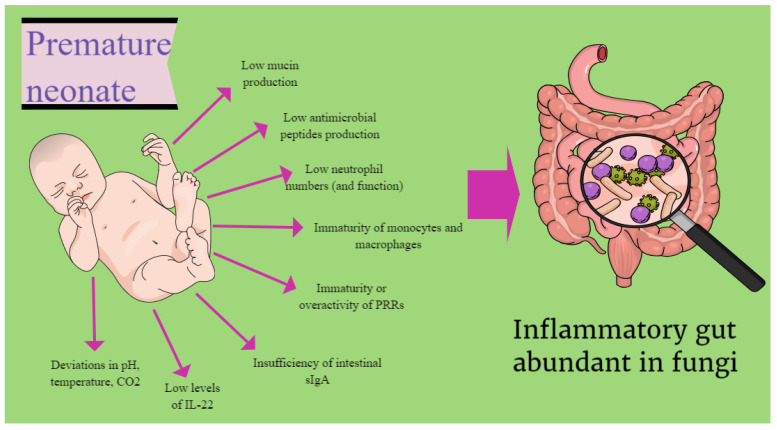
The immature immune system of premature neonates affects the composition of their gut microbiome. CO_2_: Carbon dioxide, IL-22: Interleukin-22, sIgA: Secretory immunoglobulin A, PRRs: Pattern recognition receptors.

Furthermore, low levels of IL-22 lead to an inability to limit inflammation within the gut. Moreover, in this inflammatory environment, the neonatal microbiome is abundant in fungi that are resistant to conditions associated with prematurity, such as deviations in pH, unusual temperatures, or CO2 levels (Figure 1) [47,48].

## 3. Neonatal Gut Mycobiome

Most studies on the gut microbiome are mainly related to bacterial components. They focus even more on the adult microbiome, leaving a data gap regarding the neonatal microbiome and especially the mycobiome. However, few studies have recently been published that show that the neonatal mycobiome composition and diversity are affected by both non-individual and individual factors such as climate change, the maternal mycobiome, the maternal health status and diet, the mode of delivery, the prematurity, the low birth weight, the mycobiome of breast milk, the duration of breastfeeding and hospitalization, the use of antibiotics, etc. In addition, it has been found that the neonatal gut mycobiome is related to diseases such as allergies, type 1 diabetes, and obesity, as well as gastrointestinal diseases later in life [48,49,50,51,52,53,54,55,56,57,58,59,60].

The neonatal gut mycobiome consists of a variety of fungal species prevalent in the infant’s intestinal environment. While research on the gut microbiome primarily focuses on bacterial strains, recent studies have shown that the fungal community also plays a significant role in infant gut health. The composition and structure of the gut mycobiome can be influenced by various factors, such as delivery mode, feeding practices, and antibiotic use. Further research on the neonatal gut mycobiome is essential for understanding the role of fungi in infant health and disease [55,61,62].

The adult gut mycobiome includes about 140 different genera of fungi, mainly *Candida*, *Saccharomyces*, and *Cladosporium* spp., and constitutes about 13% of the gut microbiome [63]. The gut mycobiome appears to play an important role both in digestion through the production of enzymes and vitamins and in the regulation of the immune response through the activation of fungus-specific pathogen recognition receptors (PRRs) with subsequent defense against harmful microorganisms. However, in immunosuppression but also in obesity and inflammatory bowel disease (IBD), there is a disturbance of the fungal balance in the intestine with a predominance of Saccharomycetes spp., Dipodascaceae spp., and Tremellomycetes spp. [63]. Tremellomycetes spp. were found to be associated with inflammatory conditions, while lower rates of *Saccharomyces cerevisiae* and higher *Candida albicans* were commonly seen in IBD patients. Previous studies have detected *Penicillium*, *Aspergillus*, and *Candida* species at high rates in infants up to 2 years of age. In addition, the partly vertical transmission of *C. albicans* and *Malassezia* spp. from mothers to newborns has been demonstrated. Additionally, the prevalence of *Candida* species in newborns has been shown to be 23% and appears to more than double to 50% within 4 months [63].

### 3.1. Factors Associated with Neonatal Gut Mycobiome

It has been shown that the abundance of the gut mycobiome of infants (0–2 years) and children (3–10 years) is higher than that of adults (≥18 years). It has also been found that female individuals probably have a higher abundance of mycobiome than males and present different species from them, perhaps due to the different hormones of the two sexes or due to a specific diet [64,65].

In addition, the different anatomy of the female genital organs and the proximity of the vagina to the gut may lead to greater colonization of the latter with *Candida* spp. [49].

A study demonstrated that maternal BMI and maternal diet modified the relationship between mycobiome diversity and infant BMI. It further showed that an increased abundance of fungi was associated with a lower infant BMI when, in parallel, the maternal BMI was high and the mother’s diet was unhealthy. Furthermore, it showed that during complementary feeding and after weaning, both the infant diet and the infant gut mycobiome were influenced by the maternal and paternal diets. The increase in *Saccharomyces* in the mycobiome from the age of 3 to 12 months was probably related to the introduction of solid foods [66]. Conversely, the reduction in *Saccharomyces* in infants at 12 months due to dietary changes could influence the development of obesity. In contrast, at 12 months, there was an increase in the genus *Fomitopsis*, which has antidiabetic and anti-obesity properties and has been previously associated with improved insulin sensitivity in rodent models [66,67]. A positive association between infant mycobiome with *Saccharomyces* or *Malassezia* and BMI between 1- and 5 years was also observed. Conversely, there was a negative correlation between BMI between 1 and 5 years and infant mycobiome with *Rhodotorula*. Antibiotic use between 6 and 12 months was also associated with increased fungal abundance and a lower BMI [66,67].

Moreover, previous studies have shown that early exposure to antibiotics may predispose to the development of asthma, type 1 diabetes, and inflammatory bowel disease later in life [68,69]. Arrieta et al. reported changes in early-life gut microbiota composition, with a higher mycobiome abundance than the microbiome, dominated by *Pichia kudriavzevii*, findings that were associated with the risk of later developing asthma [70].

Neonatal use of broad-spectrum antibiotics affects the composition of the gut microbiome, leading to an increase in Proteobacteria, a decrease in Bifidobacteria, and promoting commensal fungi such as *Candida* spp. to proliferate [55]. In addition, it has previously been found that cephalosporin use in premature infants can lead to invasive, systemic candidiasis, a life-threatening infection [71]. Antifungal prophylaxis, mainly with fluconazole or nystatin, is recommended in infants with predisposing factors for the development of invasive infection. It changes the composition of the gut mycobiome and prevents fungal invasion of the gastrointestinal mucosa [48]. In addition, antifungals also alter the composition and diversity of the bacterial component of the gut microbiome [72,73]. Prolonged or excessive use of antifungals and broad-spectrum antibiotics affects the microbiome and can trigger inflammatory bowel disease [74]. 

Understanding the factors that influence the neonatal microbiome, including the mycobiome, is essential for promoting infant health. These factors encompass the mode of delivery, which affects initial microbial exposure (vaginal birth vs. cesarean section); feeding practices (breastfeeding vs. formula feeding), which alter the microbiome through different nutrients and bioactive compounds; and antibiotic use, which can disrupt microbial communities and reduce diversity. Additionally, the maternal microbiome significantly impacts the neonate’s microbial colonization, while environmental exposures, such as the home environment and interactions with family members, introduce a variety of microbes. Gestational age also plays a role, as preterm infants often have distinct microbiomes, including the mycobiome, compared to full-term infants due to different medical interventions and developmental stages. Recognizing these factors (Figure 1) is critical for developing strategies to support a healthy microbiome and overall health in neonates.

### 3.2. Gestational and Postmenstrual Age and Gut Mycobiome

Schei et al. demonstrated that the detection of fungal DNA in neonatal feces has a direct correlation with the presence of detectable fungal DNA in the maternal gut and that the diversity of fungal species shows a trend to resemble the maternal mycobiome as the time interval from birth increases. Thus, during breastfeeding, *Debaryomyces hansenii* seems to prevail, while after weaning, *Saccharomyces cerevisiae* predominates. Moreover, they demonstrated that fungi can be isolated even at the age of 10 days and that *S. cerevisiae* predominates in both the maternal and neonatal guts up to 2 years of age [63]. 

As mentioned above, preterm neonates have reduced diversity in their gut microbiome. Moreover, they have an immature immune system with an incomplete intestinal epithelial barrier of increased permeability, which predisposes them to the development of nosocomial infection. Furthermore, prematurity is often associated with morbidities such as late-onset septicemia and necrotizing enterocolitis, for which prophylactic or therapeutic broad-spectrum antibiotics are administered from the very first weeks of life, a practice that predisposes to fungal predominance. In this case, there is an overgrowth of pathogenic fungi such as *C. albicans* in the gut, which, due to the immature intestinal barrier, can penetrate into the bloodstream with the subsequent development of a threatening infection. Serious fungal infection in preterm infants can also be caused by horizontal transmission of fungi such as *C. parapsilosis* from the hospital environment and medical personnel. Premature infants born at less than 27 weeks’ gestation or with a birth weight of less than 750 g who also have an indwelling central venous catheter are at a particularly increased risk of developing a fungal infection [56,63,75,76].

Gewolb et al. analyzed fecal samples from 29 extremely low birthweight infants (<1000 g) on days 10, 20, and 30 after their birth, using quantitative aerobic and anaerobic cultures. They found that there was a decrease in *Candida* gut colonization over time, from 32% at day 10 to 7% at day 30 (*p* < 0.05), and that these patterns were also similar in both breast-milk-fed and formula-fed infants [50].

A previous study attempted to shed light on how gestational age affects the gut mycobiome of preterm infants. Thus, it was shown that the gut mycobiome of premature infants consists exclusively of fungi from the genera Ascomycota and Basidiomycota, with the Ascomycota mainly of the genera *Candida*, *Davidiella*, *Debaryomyces*, *Penicillium*, and *Saccharomyces* prevailing during the first 12 months. As previously shown, apart from the gastrointestinal tract, Ascomycota and Basidiomycota dominate the mycobiome of the skin, vagina, and oral cavity [56,63,75,76]. James et al. suggested that *Candida albicans* is especially rarely found in the environment but is present in the human body, where it primarily colonizes the gastrointestinal tract [56].

Ghannoum et al. showed that *Candida* was the most prevalent genus, detected in all premature neonates, a finding that was in agreement with other studies that have shown *Candida* species, mainly *C. albicans*, to colonize both the infant and adult gut as well as the oral cavity and the vagina [51,54]. Additionally, they also identified other species of *Candida*, such as *C. metapsilosis*, *C. parapsilosis*, and *C. tropicalis*, all of which are potentially pathogenic to humans. In addition, premature infants were found to be colonized with *Candida* species at a higher rate compared to full-term infants. This study highlighted the overgrowth of *C. metapsilosis* in the gut of the most premature infants (as young as 25 weeks) who had been given prophylactic antifungal fluconazole in the first month of life. This study also showed that *Candida* continued to colonize the neonatal gastrointestinal tract for a long time after the first weeks of life, even 18 months after birth, mainly due to their long stay in the hospital due to prematurity, especially at birth ages younger than 28 weeks. In 8 out of 11 premature infants studied, *C. parapsilosis* was detected during their 12 months of life. The growth of *C. parapsilosis* was higher in most preterm infants with the youngest birth age (<31 weeks), while that of *C. albicans* was higher in those born ≥31 weeks [56].

In another recent longitudinal study, *C. parapsilosis* and *C. albicans* were both found to prevail in the rectal mycobiome of term infants in 95% and 85% of samples studied, respectively. In addition, *C. tropicalis* was found in >90% of infant samples. Both *C. tropicalis* and *C. albicans* were present at a higher rate in term samples than the rate observed in preterm samples from other studies. This was probably due to their simultaneous isolation in the vaginal samples of the mothers, meaning that the full-term babies were probably colonized during vaginal delivery [56,75].

Other yeasts that have been isolated from premature neonates are *D. hansenii*, *M. restricta*, and *S. cerevisiae. D. hansenii* is associated with dairy products and has been isolated from the skin, breast milk, and neonatal gut mycobiome during breastfeeding. *M. restricta* is a lipophilic basidiomycete that colonizes the skin of both infants and adults, so its transmission to newborns can occur either by vertical transmission from the mother or by horizontal transmission from the medical and nursing staff. *M. restricta* has also been isolated in breast milk as well as in the gastrointestinal tract.

A previous study revealed that the gut mycobiome of both preterm and full-term infants showed low diversity during the first period after birth, while as time passed, the diversity of their gut mycobiome also increased, possibly related to weaning and the gradual introduction of solid foods [52,63,77].

Other studies have shown that newborns are initially colonized by *Candida*, *Malassezia*, *Cladosporium*, and *Saccharomyces*. Around the age of 3 months, it seems that the mycobiome is dominated by *Debaryomyces*, *Candida*, *Malassezia*, and *Cladosporium*, while from the age of 1 year onwards, *Saccharomyces* dominates with a simultaneous increase in the percentage of *Rhodotorula*. This is explained by the fact that fungi that mainly colonize the skin, such as *Malassezia*, can be easily transmitted to a 3-month-old infant who is breastfed and has frequent skin-to-skin contact with the mother and caregivers. However, as it grows, it begins to crawl and feed on solid food, behaviors that predispose them to greater colonization by fungi of food or environmental origin, such as *Saccharomyces* and *Fomitopsis* [63,75,78,79,80,81,82,83].

A cohort study of children aged 0–9 years showed that a higher abundance of gut mycobiome at 2 years was associated with taller children between 2 and 9 years of age. Moreover, higher gut abundance of both fungi and bacteria and higher gut bacterial diversity at 1 year were related to lower BMI in the first year of life. This study suggested that the gut microbiome, and especially the mycobiome, can affect the development of the child, opening a field for further investigation [78].

### 3.3. Neonatal Gut Mycobiome and Short- and Long-Term Outcome 

The relationship of a healthy immune system with the composition of the gut microbiome has been demonstrated in many previous studies. It has also been hypothesized that as the diversity of the gut mycobiome increases, particularly during the first year of life, the risk of chronic disease decreases. This is particularly important in situations where the risk factor of prematurity coexists. Premature infants are born via cesarean section, leading to reduced mycobiome diversity and an increased risk for gastrointestinal comorbidities, as well as the likelihood of developing chronic diseases and allergies when they grow up [56,80,84].

Furthermore, the role of the mycobiome in the development of asthma has previously been demonstrated in murine lung models [72].

Particular situations with a large increase in *Candida* species, such as after the consumption of antibiotics, have been associated with an increase in murine lung inflammation mainly due to the production of prostaglandin E2 by *Candida* [70,85,86].

Fungal dysbiosis may be associated with gastrointestinal disease. It has been shown that patients with inflammatory bowel disease have an abundance of *Candida albicans* but less *Saccharomyces cerevisiae* in their gut mycobiome than healthy people [87,88]. In addition, an interaction between bacterial and fungal species was observed in patients with Crohn’s disease [87]. Furthermore, *Candida* appears to be more abundant in the mycobiome of T1DM and T2DM (type 1 and type 2 diabetes mellitus) patients, probably due to serum lipids (in T2DM patients) [89].

In recent research, Ascomycota, Glomeromycota, and Basidiomycota were detected by deep sequencing of biopsy specimens from adenomas and adjacent tissues. Moreover, *Phoma* spp. and *Candida* spp. dominated. Furthermore, the adenoma size and the disease stage were found to be associated with the changes in the mycobiome [90].

Recently, a correlation between reduced mycobiome diversity and obesity has been demonstrated in adults. A study showed that in the mycobiome of obese individuals, the phylum Ascomycota, the class Saccharomycetes, and the families Dipodascaceae and Saccharomycetaceae, as well as the class Tremellomycetes, predominated compared to non-obese individuals [91]. This study also supported the idea that this dysbiosis of the mycobiome in obese patients was related to changes in lipid and glucose metabolism. In contrast, the genus *Mucor* predominated in the mycobiome of non-obese individuals, likely due to its cell wall composition with chitin–chitosan, a polysaccharide that appears to have protective properties against obesity. This study also showed that the proportion of the genus *Mucor* in the mycobiome increased after weight loss in obese individuals [91].

Furthermore, *C. parapsilosis* in the mycobiome appears to promote obesity due to the production of fungal lipases that lead to an increase in free fatty acids in the gut [66,92].

## 4. Maternal Role and Other Factors Influencing the Neonatal Gut Mycobiome

**Figure 2:**
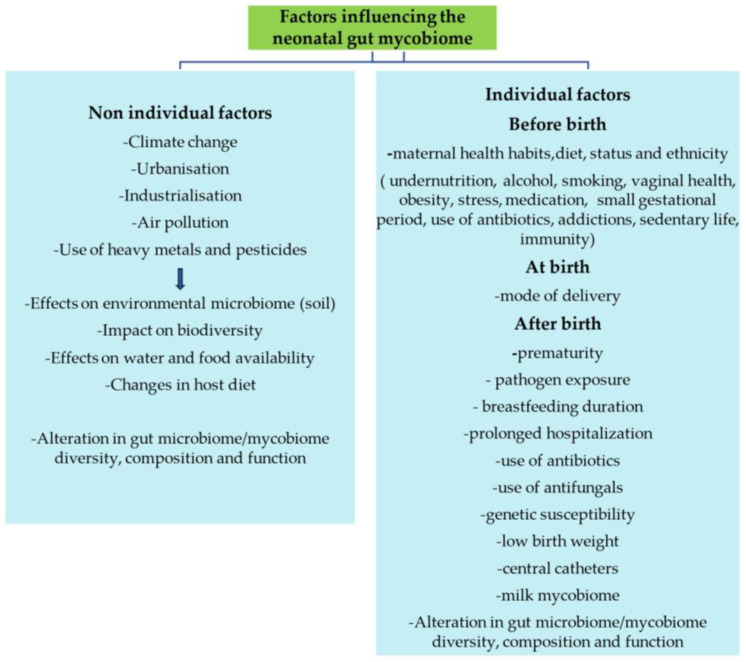
Factors influencing the neonatal gut mycobiome.

There is a wealth of data showing that the health status, habits, lifestyle, and medication of the mother affect her gut microbiome and, subsequently, her offspring microbiome (Figure 2).

### 4.1. Maternal Health Status

Maternal obesity has been associated with premature delivery (<37 weeks) and the birth of infants with neurological disorders, while gestational diabetes mellitus is associated with dysbiosis of both the maternal and neonatal gut microbiome [93,94].

Mar Rodríguez et al. analyzed the gut mycobiome in obese and non-obese individuals using Internal Transcribed Spacer (ITS)-based sequencing, demonstrating that the gut mycobiome seems to be affected in obese patients, a finding associated with changes in lipid and glucose metabolism [91]. They found that the two major phyla, Ascomycota and Basidiomycota, were not significantly different between obese and non-obese individuals, while the phylum Zygomycota was almost absent among obese patients. The class Tremellomycetes appeared only in obese individuals, while the class Agaricomycetes had a higher abundance in non-obese patients. Aspergillaceae and Mucoraceae were the most prevalent families in non-obese individuals, while Dipodascaceae, Aspergillaceae, and Saccharomycetaceae were the most abundant in obese patients. *Candida*, *Nakaseomyces*, and *Penicillium* were the most abundant genera detected in obese patients, while *Mucor* was the most prevalent genus in non-obese patients, followed by *Candida* and *Penicillium*. Furthermore, this low abundance of the genus *Mucor* in obese patients was unexpectedly corrected during weight loss [91].

Kowalewska et al. showed that children and adolescents with T1DM had a mycobiome with greater species diversity and a lower prevalence of *Candida albicans* compared to control subjects. Interestingly, fungal species in T1DM patients showed higher resistance to antifungal therapy [53].

Hoffmann et al. analyzed archaea and fungi by deep sequencing of marker genes in DNA purified from fecal samples and found 66 fungal genera, dominated by Ascomycota or Basiodiomycota. Furthermore, they showed that *Methanobrevibacter* and *Candida* were mainly associated with diets rich in carbohydrates but not with diets rich in amino acids, proteins, and fatty acids. High *Candida* abundance was mainly associated with recent carbohydrate consumption, while *Methobrevibacter* abundance was associated with both long-term and recent carbohydrate consumption [95].

Gouba et al. analyzed the gut microbiome of an anorexic patient using both culture and PCR sequencing. Ten different fungal species were detected, including *S. cerevisiae*, *A. flavus*, *M. pachydermatis*, *M. globose*, and *M. restricta*, as well as *C. capitatum*, *Sclerotium* sp., *A. ruber*, *P. solitum*, *C. bruhnei*, and *Tetratrichomonas* sp. Most of these fungi were associated with the patient’s diet (cereals, fruits, seafood, etc.). The authors found that the diversity of fungal species in the anorexic patient was lower than they had found in an obese patient in a previous study [96].

Nash et al. found that in the human gut mycobiome, there is a yeast prevalence, mainly *Saccharomyces*, *Malassezia*, and *Candida* [76].

Maternal obesity also modulates breast milk composition. In addition, as obesity is associated with inflammation and oxidative stress, it affects the synthesis of immune-related components in breast milk, such as immunoglobulins, lactoferrin, leptin, ghrelin, adiponectin, C-reactive protein, growth factors, extracellular vesicles, and lymphocytes. Studies have shown that this not only affects the newborn’s immune system immediately after birth but also possibly regulates the infant’s health over a long period of time [97]. Breast milk contains compounds that affect and modulate the infant’s immune system, such as human milk oligosaccharides (HMOs) produced in the mammary gland from lactose, which inhibit the adhesion of microorganisms to the intestinal mucosa, the production of bacteriocins and organic acids, the expression of genes involved in inflammation, and moreover, increase the functioning of the intestinal barrier [98,99].

However, breast milk is not sterile but contains microorganisms, which, through breastfeeding, pass to the infant and contribute to the formation of the offspring gut microbiome [63]. This human milk microflora can be affected by breast pathology, mode of delivery, antibiotic use, maternal health, and gestational age [100,101]. Maternal habits such as diet, medication, lifestyle, addictions, mental health, etc. can also affect the nutrients in breast milk [57,58].

The composition of human colostrum (HC), the initial form of mother’s milk, is uniquely tailored to meet the specific requirements of each neonate, particularly premature infants. It provides abundant proteins and immunomodulating components, which contribute to shielding against necrotizing enterocolitis (NEC) and the risk of late-onset septicemia. These protective effects may arise, among others, from its elevated levels of lactoferrin [102,103]. 

Other studies have indicated an association between infant sex and the composition of the milk microbiome, breastfeeding patterns, exclusivity, and lactation stage. Additionally, there is a hypothesis that the milk microbiome could originate partly from the infant’s oral microbiome, which is believed to differ between sexes. It has also been hypothesized that the milk microbiome could originate partly from the infant’s oral microbiome, which is believed to differ between sexes. Moreover, differences in milk microbiome between sexes are likely influenced by other sex-dependent differences in other milk components such as calcium, cortisol, and fat. In addition, mainly hormone-dependent differences in the gut microbiome between sexes have been found [101].

To the best of our knowledge, very few studies have attempted to analyze the mycobiome of breast milk. Boix-Amoros et al. demonstrated the universal presence of fungi in breast milk, arguing that the initial spread of fungal species in the neonatal gut may derive in part from the consumption of breast milk. In this study, Basidiomycota and Ascomycota were found to prevail, and especially *Malassezia* and *Davidiella* were found to be the dominant genera on all continents. In addition, a prevalence of different species of fungi was observed in association with the mode of delivery. Thus, Ascomycota species, *Cryptococcus*, and *Candida smithsonii* prevailed in milk samples from women after vaginal delivery, while *Sistotrema*, *Malassezia restricta*, and *Davidiella tassiana* prevailed in milk samples after cesarean birth [52].

Heisel et al. demonstrated that both neonatal intensive care unit (NICU) surfaces and maternal breast milk were colonized by different fungal species, mainly *Candida* and *Saccharomyces* species, which could affect the neonatal gut mycobiome. In their study, the samples were analyzed by sequencing the Internal Transcribed Spacer region 2 (ITS2) of the fungal rDNA locus, and they found that the breast milk samples had a higher fungal diversity than those obtained from the surfaces of the neonatal unit. From the analysis of the samples, *Candida albicans*, *Candida parapsilosis*, and *Saccharomyces cerevisiae* were found in abundance. Moreover, 21 fungal species were isolated in greater abundance in breast milk compared to NICU surfaces, including *Candida glabrata*, *Candida tropicalis*, and *Cryptococcus neoformans* [59].

Schei et al. hypothesized that the *Debaryomyces hansenii* they detected in the neonatal gut mycobiome possibly came from the breast milk, as this was the exclusive food until the age of 3 months of the newborns who participated in their study [63]. Moreover, in another previous study, *Candida* (*Candida parapsilosis*), *Saccharomyces*, and *Rhodotorula mucilaginosa* were also found in the human breast milk of healthy mothers [77].

Preeclampsia often leads to preterm birth and poor neonatal outcomes, such as hypoxic–ischemic encephalopathy, respiratory distress syndrome, and neonatal retinal hemorrhage [104]. Although the exact etiology of preeclampsia is unknown, four main mechanisms have been suggested to be involved, such as oxidative and endoplasmic reticulum stress, platelet and thrombin activation, endovascular inflammation, and endothelial dysfunction. Previous studies have shown that the gut microbiome of mothers could influence these mechanisms [105,106]. Hua Zou et al. attempted to understand the association of the maternal mycobiome with preeclampsia. Although fungi occupy only 01%–1% of the gut microbiome, they present a high diversity of more than 50 species from genera such as *Candida*, *Aspergillus*, *Cryptococcus*, *Malassezia*, *Cladosporium*, and *Trichosporon*. As has already been demonstrated, cells of the immune system, such as dendritic cells, macrophages, and natural killer T cells, can recognize, through their pattern recognition receptors (PRRs), carbohydrate components found in fungal cell walls such as glucan or mannan [107]. Hua Zou et al. found that in fungal dysbiosis, TLR receptors on dendritic cells, mainly TLR2 and TLR4, recognize the associated antigens and activate the MYD88 or TRIF pathways to secrete IL-23, IL-1β, and IL-6, resulting on the one hand in the response of immune-assisted T1 and Th17 immune cells and on the other hand in IL-6-mediated inhibition of the immune modulation by Treg cells. IL-6 can also inhibit the differentiation of Treg cells, causing an imbalance of Treg/Th17 immune cells, which is one of the main mechanisms in the pathogenesis of preeclampsia [108,109]. Hua Zou et al. also demonstrated that the gut mycobiome changed significantly in the third trimester of pregnant women with preeclampsia, mainly with a predominance of Ascomycota. They also showed that choline metabolism was closely related to fungi and that Ascomycota, particularly *Candida* spp., could affect the percentage of glycerophosphocholine in the gut mycobiome and therefore the choline metabolism, triggering the onset of preeclampsia in pregnant women [109].

### 4.2. Maternal Diet and Medication

Any factor, exogenous (such as maternal diet and medication) as well as endogenous (such as maternal stress and changes in her metabolic state), could affect the maternal gut microbiome with consequences for fetal development [110]. Gutierrez M.W. et al. also demonstrated that maternal factors combined with mycobiome maturation patterns may influence growth dynamics in the first 5 years of life [66]. In particular, the use of broad-spectrum antibiotics during pregnancy reduces diversity in the mother’s gut microbiome and may trigger an early onset of inflammatory bowel disease in the child [111]. It has also been suggested that supplementation of the maternal diet with probiotics leads to a higher abundance of total fungal DNA and a lower abundance of *S. cerevisiae* in the maternal gut [84]. 

It has been shown that the composition of the gut mycobiome in pregnant women changes from early to late pregnancy, to a greater extent compared to changes seen in gut bacteria. In addition, the gut mycobiome appears to be related to host metabolism as well as pregnancy health and outcome [112]. 

A recent study showed that the placental microbiome of pregnant women was correlated with their vaginal and gut microbiomes only at 32–34 weeks of pregnancy. Moreover, the vaginal microbiome at full term was unrelated to the placental microbiota. In addition, the gut microbiome had a negative correlation with the placental microbiome [113].

Recent studies have demonstrated the presence of a bacterial microbiome in the placenta, even in cases of term and uncomplicated pregnancies [63,114]. These findings suggest the potential for bacterial metabolite transfer from the mother’s gut microbiome to the fetus through the placenta, thereby influencing its development [115]. 

Delivery mode may also affect neonatal colonization. As it has been previously demonstrated, *Candida* species are mainly vaginally transmitted to babies who are naturally born, while *Malassezia* is predominantly found in babies born by cesarean section delivery, possibly from the maternal skin [62]. However, in one study, *Trichosporon* was the dominant genus in cesarean-delivered neonates [61].

Dinleyici et al. reported that *Saccharomyces cerevisiae* predominated in maternal milk on days 7–15 after cesarean birth, followed by *Aspergillus glaucus*, but disappeared on days 45–90, when *Penicillium rubens* increased. On the contrary, *Malassezia globosa* predominated all milk samples after vaginal deliveries [116].

### 4.3. Climate Change and Geographic Location

Climate change can also affect the maternal total gut microbiome. The abundant production of carbon dioxide (CO_2_) leads to changes in the composition of the soil and the nutrients in the food, causing disorders of the gastrointestinal system [117,118]. In addition, the use of heavy metals, pesticides, and synthetic fungicides in agriculture can reduce the diversity of the environmental microbiome and affect the availability of food and water. In addition, dietary consumption of synthetic fungicides can have detrimental consequences for human health and the development of resistance to fungal species [118]. Climate change also causes thermal toxicity in epithelial cells, thereby affecting the intestinal epithelial barrier, with serious consequences for a developing immune system as it makes it vulnerable to infections, atopy, autoimmune diseases, and chronic gastrointestinal inflammation [119]. 

Furthermore, climate change causes severe weather disasters. A recent study found that prenatal maternal hurricane exposure was associated with reduced microbial diversity and altered offspring gut metabolic capacity [120]. In addition, the different climatic conditions around the world particularly affect the growth of specific species of fungi to which individuals may be exposed and which may determine the composition of their mycobiome [121].

Air pollution and increasing levels of particulate matter (PM) can also cause inflammation, oxidative stress, and the development of serious human diseases. Such environmental factors may lead to food insecurity, resulting in the overconsumption of overly processed unhealthy foods and the subsequent development of malnutrition, obesity, and changes in the gut microbiome [119]. Moreover, atmospheric pollution can result in a lack of outdoor exercise, a sedentary lifestyle, obesity, and changes in the gut microbiome as well [119,122,123].

Santoyo et al. showed that children living in an industrialized urban environment in Mexico City presented changes in their gut microbiome similar to those of children infected with *Ascaris lumbricoides* from a rural indigenous population in the remote mountainous region of Guerrero, Mexico [124]. Urbanization appears to negatively affect the diversity of the human microbiome. 

Nakayama et al. showed that *Bacteroides* predominated in the microbiome of adolescents living in cities, while *Prevotella* predominated in those living in rural areas. They also showed that the microbiome of people from the West had an abundance of *Bacteroides* due to their diet of more high-fat meat, while those living in the East had an abundance of *Prevotella* due to their diet of more high-fiber carbohydrates [125]. 

Furthermore, another study showed that the composition of the gut microbiome differed among adolescents living in different urban areas and was influenced by factors such as dietary habits, taste preferences, sleep duration, and exercise [126]. 

Kabwe et al. demonstrated that the gut mycobiome can be influenced by geographic location, mainly due to climatic conditions, which can affect agriculture and dietary habits. They further evaluated, using Internal Transcribed Spacer (ITS) gene analysis, biomarker species specific to rural and urban cohorts. They found a predominance of Pichia, followed by *Candida* and *Cladosporium.* The relative abundance of *Pichia* was higher in rural areas compared to urban locations. Moreover, the relative abundance of *Candida* and *Cladosporium* was higher in urban areas compared to rural areas. They further showed that factors such as smoking, mode of birth, and breastfeeding also determined the gut mycobiome composition [127]. Morandini et al. compared the gut microbiomes of 60 mother–infant pairs from rural and urban areas of Senegal and found that the microbiome of urban mothers, who were also more often overweight, differed from those living in rural areas and showed a predominance of Lachnospiraceae and *Enterobacter.* In addition, their infants showed delayed maturation of the gut microbiome and a higher susceptibility to infectious diseases [128]. 

Kortman et al. demonstrated differences among five regions in Vietnam in both breast milk composition and infant gut microbiota, possibly due to maternal diet. For example, in the Ha Noi region, an abundance of Enterobacteriaceae was observed in both breast milk and the gut microbiome of infants. The study authors suggested that the high iron content also found in breast milk in Ha Noi may have promoted the growth of Enterobacteriaceae. However, they were unable to pinpoint the exact source of the iron, hypothesizing that it was either due to higher maternal iron intake formulas or in her diet rich in meat, or even due to the polluted groundwater around Ha Noi [129].

In other studies, *Malassezia* was found in lower abundance and *Rhodotorula* and Saccharomycetales in higher abundance in South African breast milk samples; *Penicillium* and *Rhodotorula* were detected in lower abundance, while *Malassezia* was found in higher abundance in samples from China; and *Saccharomyces* was found in high abundance in the Spanish and Finnish samples. In general, *Malassezia*, *Davidiella*, *Sistotrema*, and *Penicillium* genera were detected in milk samples from China, Spain, Finland, and South Africa [52].

To the best of our knowledge, most studies show how factors such as diet, lifestyle, and geography can shape the human gut microbiome, focusing mainly on the bacterial gut component. Few studies have evaluated factors that may also influence the gut mycobiome, including age, sex, diet, diabetes, obesity, malnutrition, smoking, alcohol, and geographic locations [50,130]. 

Strati et al. investigated the gut mycobiome of 111 healthy individuals by culture on fungal selective media and by amplicon-based ITS1 metagenomics analysis on a subset of 57 individuals. They also studied the tolerance to the gastrointestinal (GI) environment and the susceptibility to antifungals of the isolated fungi. A total of 34 different fungal species were isolated with different tolerances to gut conditions such as body temperature (37 °C), acidic and oxidative stress, and bile salt exposure [49]. In addition, they observed that half of the isolates had the ability to form hyphae or pseudohyphae, which are mechanisms of adhesion and penetration into the gastrointestinal mucosa. They suggested that *C. albicans*, which withstands the adverse conditions of the intestinal environment and colonizes it under normal conditions, can easily become a pathogen due to its ability to transform itself by forming pseudohyphae and hyphae. It was also interesting that in this study, the fungi isolated showed a high frequency of resistance to azoles, a finding with particular clinical significance [49].

Young-Do Nam et al. analyzed the fecal samples from ten Koreans by using the PCR-fingerprinting method and denaturing gradient gel electrophoresis (DGGE) and found that the bacteria, mainly Firmicutes and Bacteroidetes, predominated, followed by Archaea and only a small number of fungi and *Blastocystis hominis* [131].

Hamad et al. analyzed a stool sample from a healthy African man using culture-dependent and extensive molecular methods targeting 18S rRNA and ITS sequences, finding that very few fungi, mainly *Candida* spp., *Galactomyces* spp., and *Trichosporon asahii*, were isolated using culture-based methods, while the majority of fungi required other culture-independent methods [132].

Other studies examined the gut mycobiome diversity in poor cities in rural Africa and revealed that Ascomycota and Basidiomycota dominated the gut mycobiome of South African infants. In addition, *M. globosa* was found to predominate in infants living in rural communities in Ghana during the first 3 months of life [133].

## 5. Conclusions

In conclusion, the neonatal gut mycobiome refers to the community of fungi residing in the gastrointestinal tract of newborns. This microbial ecosystem, predominantly composed of various fungal species, plays a significant role in early-life gut health and development. The composition and diversity of the neonatal gut mycobiome can be influenced by several factors, including mode of delivery, feeding practices, antibiotic exposure, and maternal health status. Emerging research suggests that disturbances in the neonatal gut mycobiome may be linked to a range of health conditions, including allergies, autoimmune diseases, obesity, and gastrointestinal disorders later in life. Understanding the intricate dynamics of the neonatal gut mycobiome is essential for unraveling its role in human health and disease and for developing targeted interventions to promote optimal gut health from infancy through adulthood. Absolutely, there is a clear need for further studies in this area. Expanding research on the neonatal gut mycobiome could provide valuable insights into its role in health and disease across the lifespan. These studies could focus on elucidating the mechanisms by which the neonatal gut mycobiome influences health outcomes, exploring the impact of various factors related to maternal and neonatal status on its composition, and investigating potential therapeutic interventions to modulate the gut mycobiome for improved health outcomes. By addressing these knowledge gaps, we can deepen our comprehension of the neonatal gut mycobiome and its significance for human health, thus laying the groundwork for future interventions that are both targeted and efficacious.

## Data Availability

Data are contained within the article.
